# Higher Incidence of Lung Adenocarcinomas Induced by DMBA in Connexin 43 Heterozygous Knockout Mice

**DOI:** 10.1155/2013/618475

**Published:** 2013-10-03

**Authors:** Krishna Duro de Oliveira, Marcello Vannucci Tedardi, Bruno Cogliati, Maria Lúcia Zaidan Dagli

**Affiliations:** Department of Pathology, School of Veterinary Medicine and Animal Science, University of São Paulo, Avenue Professor Dr. Orlando Marques de Paiva 87, 05508-900 Sao Paulo, SP, Brazil

## Abstract

Gap junctions are communicating junctions which are important for tissue homeostasis, and their disruption is involved in carcinogenic processes. This study aimed to verify the influence of deletion of one allele of the Connexin 43 gene on cancer incidence in different organs. The 7, 12-dimethylbenzanthracene (DMBA) carcinogenic model, using hebdomadary doses by gavage of 9 mg per animal, was used to induce tumors in Connexin 43 heterozygous or wild-type mice. The experiment began in the eighth week of the mice life, and all of them were euthanized when reaching inadequate physical condition, or at the end of 53 weeks. No statistical differences occurred for weight gain and cancer survival time (*P* = 0.9853) between heterozygous and wild-type mice. Cx43^+/−^ mice presented significantly higher susceptibility to lung cancer (*P* = 0.0200) which was not evidenced for benign neoplasms (*P* = 0.3449). In addition, incidence of ovarian neoplasms was 2.5-fold higher in Cx43^+/−^ mice, although not statistically significant. Other organs showed a very similar cancer occurrence between Cx43 groups. The experiment strengthens the evidence of the relationship between Connexin 43 deficiency and carcinogenesis.

## 1. Introduction

Gap junction, first described in the beginning of the 1960's [1], is a cell membrane junction responsible for cell-to-cell communication, being one of the mechanisms involved in tissue homeostasis, proliferation, and differentiation [2–5]. Connexins are the basic units of these junctions, being expressed by 21 genes in human and 20 in mice [6]. The union of 6 of these proteins is responsible to form a connexon, a hemichannel structure normally allocated in cell membrane [5, 7, 8]. When connexons from neighbor cells connect, they allow the interchange of substances with 1,000 Da or less, like water, ions, sugars, small peptides, amino acids, fatty acids, and drugs [5, 9]. 

Cell growth, proliferation, and apoptosis are regulatory mechanisms shared between cells, and any disruption of this process may allow the development of many disorders. Cell-to-cell communication pathway has been studied to better understand inflammatory, regenerative, and oncogenic processes [3, 7, 10]. A considerable number of studies after the classic Loewenstein and Kanno [11] publication have shown the correlation between the lower communication capacity and the development of cancer. 

This study has been performed in order to verify if the heterologous deletion of one allele of Cx43 could contribute to enhance the incidence of different cancers in mice, when compared to wild-type mice. DMBA carcinogenesis was the model of choice, based on previously conducted experiment, where breast, lung, skin, lymphoid, digestive tract, and ovary cancers were evidenced [12]. 

## 2. Materials and Methods

### 2.1. Animals

The heterozygous knockout mice for Connexin 43 (Cx43^+/−^) were generated by replacing exon-2 of the Cx43 gene by neomycin resistance gene [13]. This animal model was used because Connexin 43 knockout mice (Cx43^−/−^) die after birth due to cardiac congenital abnormalities [13]. Animals were kindly provided by International Agency for Research on Cancer (IARC, Lyon, France) and originally were produced in the C57BL/6 strain. Their background was subsequently changed to CD1 by serial breeding at the IARC. The Cx43 expression was characterized by real-time polymerase chain reaction (PCR) and Western blot in these Cx43^+/−^ mice, showing reduced mRNA amount and lower Cx43 levels than wild types according to previouse studies from our group [14]. 155 female mice wild-type (Cx43^+/+^) and heterozygote (Cx43^+/−^) mice were randomly provided by the Animal Facility of the Department of Pathology, School of Veterinary Medicine and Animal Science of the University of São Paulo. The experiment was blind; the authors were not informed if animals were wild types or heterozygotes during the mice life time, necropsy, and histopathology procedures. Only female mice were used to favor breast cancer development. 

The animals were kept in a room with ventilation (16–18 air changes/hour), relative humidity (45–65%), controlled temperature (20–24°C), and light/dark cycle 12:12 and were given water and balanced diet *ad libitum*. The study has been approved by the Committee on Bioethics of the School of Veterinary Medicine and Animal Science of the University of São Paulo, Proc. no. 1876/2010.

### 2.2. Carcinogenesis

Carcinogenesis was induced by f 7, 12-dimethylbenzanthracene (Sigma), diluted in corn oil and administered by gavage [12]. Each animal received 1 mg per week until completing the total dose of 9 mg. Control group was composed of 20 mice, receiving only corn oil, also by gavage. Animals were weighted and received careful clinical examination weekly. The experiment began in the eighth week of life of animals, and all animals were euthanized when reaching inadequate physical condition, or at the end of 53 weeks.

### 2.3. Necropsy and Histopathology Study of Tumors

Necropsy was performed in all animals of the experiment, and tumors, mammary glands, gastrointestinal tract, liver, spleen, heart, lung and kidneys were collected and fixed in 10% formaldehyde solution. All mouse tumors were classified according to IARC Scientific Publication no. 111 [15].

### 2.4. Genotyping

DNA from each mouse was obtained from tail biopsies. Mice were genotyped for Connexin 43 gene by polymerase chain reaction (PCR) as described by Yamakage and collaborators (1998) [16]. The primers were used in respect to the following sequence: CCCACTCTCACCTATGTCTCC-3′ and antisense 5′-ACTTTTGCCGCCTAGCTATCCC-3′ observed at 520 bp; neo-sense 5′-GGCCACA GTCGATGAATCCAG-3′ and antisense 5′-TATCCATCATGGCTGATGCAA-3′ observed at 294 bp.

### 2.5. Statistical Analysis

Statistical analysis was performed using the GraphPad Prism (version 5.0, GraphPad Software Inc., USA). Chi-square test and Fisher's exact test were used to compare incidence between both Cx43^+/−^ and Cx43^+/+^ groups. Odds ratio and relative risk and descriptive studies of the mean of tumors per animal in each group were also assessed. Logrank test was performed in order to evaluate the survival difference between Cx43^+/+^ and Cx 43^+/−^ mice. The significance level was set at *P* < 0.05. 

## 3. Results

Control group after genotype was composed of 10 homozygote animals (wild types) and 10 heterozygous animals for Cx43, and none of them developed any type of neoplasia. 135 mice that received DMBA after genotype were divided in two groups, composed of 60 wild-type mice and 75 Cx43 heterozygous mice. 

Neoplasms began to appear in the 8th week after DMBA injection and affected 100% of DMBA treated mice. 

**Figure 1:**
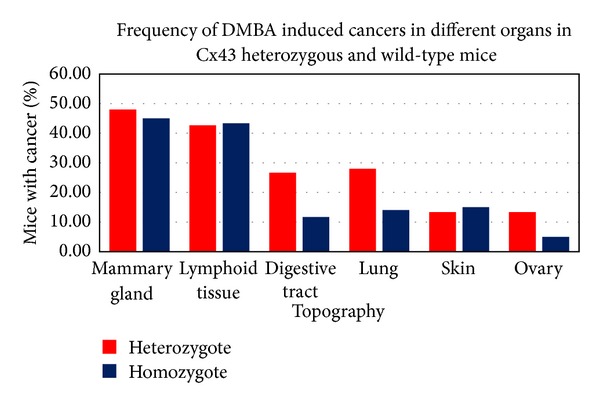
Bar graphic showing the proportional incidence among different topographies compared with the Cx43^+/−^ and Cx43^+/+^ groups.

No significant statistical differences were observed in weight gain and survival time (*P* = 0.9853) between Cx43^+/−^ and Cx43^+/+^ DMBA induced groups (data not shown). Incidence rate of neoplasms in different organs and the histopathological classification of the neoplasms are presented in Table 1 and Figure 1.

Chi-square test and Fisher's Exact test were performed to the observe difference in incidence of cancers in different locations between Cx43^+/−^ and Cx43^+/+^ DMBA induced groups. 

**Figure 2:**
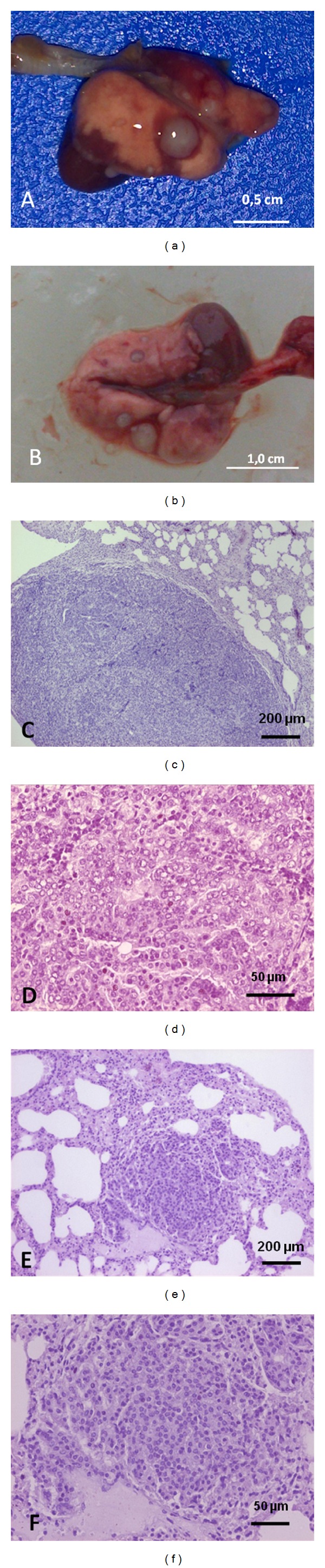
(a) Gross picture of Cx43^+/−^ mouse lung, showing gray nodules of 0.1–0.3 cm of diameter. (b) Gross picture of Cx43^+/+^ mouse lung, showing red and grey nodules of 0.3 cm or less of diameter. Bar = 0,5 cm, and bar = 1,0 cm. (c) and (d). Lung bronchial carcinoma, H&E, bar = 200 *μ*m, and bar = 50 *μ*m. (e) and (f). Lung with solid papillary adenomas, H&E, 200 *μ*m, and 50 *μ*m.

It has been detected a statistically significant difference in the incidence of lung adenocarcinomas, where Cx43^+/−^ mice presented a 1.4-fold higher risk than wild-type animals (Table 2). Histopathology images of lung cancer are presented in Figure 2.

Benign lung neoplasia was diagnosed in 8 and 3 animals from Cx43^+/−^ and Cx43^+/+^ DMBA treated animals, respectively. The relative risk for developing papillary lung adenoma in heterozygous group, when compared with wild-type animals, was 2.133 (IC 95% 0.5913–7.696), and odds ratio were 2.269 (IC 95% 0.5745–8.959). The *P*  value was 0.3449 for Fisher's Exact Test, and therefore, there was no difference in the incidence of lung papillary adenomas in Cx43^+/−^ and Cx43^+/+^ mice.

## 4. Discussion

The aim of this study was to verify if the deficiency in Connexin 43 could increase the susceptibility to different types of neoplasms in mice. For this purpose, the DMBA carcinogenesis model was used, since it has previously been shown, in BALB/c mice, that this carcinogen induces not only mammary tumors but also lung, digestory, lymphoid, and other neoplasms [12].

The 100% of cancer occurrence in DMBA induced mice in this study is in accordance with the incidence rate of cancers in the study presented by Tedardi et al. [12]. The purpose was to investigate possible correlations of the deletion of one Cx43 allele on cancer development in different organs. The statistical analysis demonstrated a higher incidence of lung cancer in Cx43^+/−^ mice when compared to wild-type animals.

Gap junctions are known to be involved in lung carcinogenesis. This information can be clearly demonstrated by Cesen-Cummings et al. study [17]. They cultivated human and mouse cell lines of normal and neoplastic lung tissues and compared the cell-to-cell transfer of Lucifer Yellow coupling dye, observing a higher dye transfer in nonaffected tissue than in cancerous ones (represented by lung small cell carcinoma, squamous cell carcinoma, adenocarcinoma and large cell carcinoma in human cell lines and carcinoma in mouse cell line) [17]. Coculturing the normal and cancer cells, they still noted the lower capacity of neoplastic cells to communicate with each other and with normal cells [17]. 

Several studies evidenced the correlation of Cx43 expression in lung carcinogenesis. Human and mouse lung cancer cell lines had a lower expression of connexin 43 by Western blot and Southern blot analysis [17] and immunohistochemistry. In a study with 107 samples of human lung cancers, it has been shown a decrease in numbers of Cx43 spots and loose of membrane stain with replacement by cytoplasm subcellular localization of connexins [18]. The relation of decreased expression of both Connexin 43 and E-cadherin was associated with a poor differentiation, advanced TNM stage, and lymph node metastasis [18]. The expression decay occurred progressively from normal distant tissue to adjacent tissue and cancer nodules and is related to nodal lung micrometastasis [19]. Connexin 43 expression also correlated with the cancer degree of differentiation. It has been shown that poorly differentiated lung adenocarcinoma and squamous cell carcinoma expressed lower levels than well-differentiated and moderate-differentiated cancers [20]. This aberrant expression could be explained by promoter methylation, probably for AP1 binding, [19].

Higher incidence of lung tumors was initially described by our research group. Heterozygous knockout mice Cx43^+/−^ presented statistically more lung papillary adenomas and with a higher number of cells stained for PCNA than wild-type animals. Furthermore, the lesions had larger areas, and these animals presented lower expression or Cx43 mRNA [14]. This experiment used urethane induction, and mice lived only 6 months; maybe the short experiment duration and the different carcinogen used could explain why we found correlation with malignant tumors and not with benign ones. 

Another study from our group using NNK for lung carcinogenesis induction corroborates the Cx43^+/−^ susceptibility to spontaneous and induced lung cancer and demonstrates that in the heterozygous mice, nodules were larger and surprising expressing higher levels of Cx43 mRNA [21]. Lung neoplasms, spontaneous, or chemical induced are known to be generated by alveolar type II epithelial cells (APTIIs) [22, 23]. Cx43^+/−^ animals had a lower cell-to-cell communication capacity and elevated proliferation of APTIIs [22], and the transfection of *Gja1 *(Cx43 gene) gene in E9 APTII neoplasia cells, in other study, reestablished and rendered these cells to a nonneoplastic state [23]. The role of Cx43 must be extensively studied for molecular carcinogenesis processes comprehension in the lung. 

Another point to highlight is the ovarian cancer occurrence in this experiment. It is known that the connexin 43 is expressed in ovarian tissue, being governed by gonadotropins in transcription, translation, and posttranslational modification. Within their physiological functions, Cx43 plays a role in the control of follicular genesis and oogenesis and seems to act here as a tumor suppressor gene [24]. According to Fernstrom et al. (2002) [25], gap junction proteins are often reduced in neoplastic cells, including cells of ovarian carcinoma. The same group of researchers conducted an interesting study *in vitro* to gene therapy, using the Cx43 directed to ovarian cancer. Transfecting ovarian carcinoma cells with Cx43 gene decreased cell proliferation and increased sensitivity to adriamycin, suggesting that communication by gap junction and/or Connexin 43 is able to suppress the neoplastic phenotype of ovarian carcinoma cells and its low expression is involved in neoplastic transformation of these cells. Although our observations in this paper have not found statistically significant differences in values between the groups, in absolute numbers, in the group with heterologous deletion of Cx43, there was a higher incidence of ovarian neoplasms, above twice than manifested in wild group. It is possible that if a larger number of animals were studied, the correlation would demonstrate what strongly corroborate with this role assigned to Cx43, whose depletion could favor the ovarian carcinogenesis.

## 5. Conclusion

The Cx43 deficient mice predisposition to lung neoplasms had been demonstrated as well as the gap junction role in the carcinogenic process in this organ. Our study strengthens the evidence by DMBA carcinogenic protocol, which is not a lung-specific carcinogen. In addition, the predisposition of ovary granulosa cell carcinoma for Connexin 43 deficiency had a consistent literature basement, and the authors suggest further studies to correlate both *in vivo* in a more specific carcinogenic model for this kind of tumor.

## Figures and Tables

**Table 1:** DMBA induced cancer incidence in Cx43^+/−^ and Cx43^+/+^ mice divided by topography and tumor histopathological classification.

Cancer	Cx43^+/−^ (*n* = 75)		Cx43^+/+^ (*n* = 60)	
	Number of tumors	%	Number of tumors	%
*Mammary gland *	*n* = 36	48.00	*n* = 27	45.00
Adenoacanthoma	20	47.62	10	34.48
Adenocarcinoma type A	5	11.90	4	13.79
Adenocarcinoma type C	1	2.38	0	0.00
Carcinosarcoma	0	0.00	0	0.00
Cystic adenocarcinoma	12	28.57	12	41.38
Fibrosarcoma	1	2.38	0	0.00
undifferentiated type	1	2.38	0	0.00
Organoid	2	4.76	0	0.00
Total	**42**	**100.00**	**29**	**100.00**

*Digestive tract *	*n* = 20	26.67	*n* = 16	26.67
Squamous cell carcinoma	20	95.24	16	5.52
Gastric adenocarcinoma	1	47.61	0	0.00
Total	**21**	**100.00**	**16**	**100.00**

*Lymphoid tissue *	*n* = 32	42.67	*n* = 26	43.33
Lymphoma	32	100.00	26	100.00
Total	**32**	**100.00**	**26**	**100.00**

*Skin *	*n* = 10	13.33	*n* = 9	15.00
Squamous cell carcinoma	10	100.00	9	100.00
Total	**10**	**100.00**	**9**	**100.00**

*Lung *	*n* = 21	28.00	*n* = 7	11.66
Papillary adenocarcinoma	21	95.45	7	100.00
Bronchial carcinoma	1	4.45	0	0.00
Total	**22**	**100.00**	**7**	**100.00**

*Ovary *	*n* = 10	13.33	*n* = 3	5.00
Granulosa cell carcinoma	10	100.00	3	100.00
Total	**10**	**100.00**	**3**	**100.00**

Total	**137**		**90**	
Number of cancer histological types per animal	1.83		1.50	

Note: there are more tumors than mice in the experiment because some animals developed more than one type of cancer in the same or different organs.

**Table 2:** Cancer incidence in DMBA wild-type and heterozygote mice induced groups.

Cancer topography	Heterozygote (Cx43^+/−^)	Wild-type (Cx43^+/+^)	Odds ratio (95% IC)^1^	Relative risk (95% IC)^1^	*P* value
Mammary gland	36	27	1.128	1.067	0.7285
			(0.5708−2.230)	(0.7399−1.538)	
Lymphoid tissue	32	26	0.9732	0.9846	0.9380
			(0.4902−1.932)	(0.6662−1.455)	
Digestive tract	20	16	1.000	1.000	1.0000
			(0.4640−2.155)	(0.5694−1.756)	
Lung	21	7	2.944	2.400	0.0200*
			(1.155−7.506)	(1.094−5.264)	
Skin	10	9	0.8718	0.8889	0.7820
			(0.3296−2.306)	(0.3859−2.047)	
Ovary	10	3	2.923	2.667	0.1436
			(0.7664−11.150)	(0.7678−9.262)	
Total	**75**	**60**	**—**	**—**	**—**

^1^Values calculated using wild-type mice (Cx43^+/−^) cancer incidence for reference to compare with cancer incidence with heterozygote group (Cx43^+/+^).

*Results of *P* value less than 5%.
